# Association between metabolic activity of visceral adipose tissue and retinal vein occlusion: a preliminary ^18^F-FDG PET/CT study

**DOI:** 10.3389/fendo.2025.1679216

**Published:** 2025-10-02

**Authors:** Kwang-Eon Choi, Chanmin Joung, Eungyu Yoon, Hyun Woo Chung, Ki Joo Pahk, Kisoo Pahk

**Affiliations:** ^1^ Department of Ophthalmology, Korea University College of Medicine, Seoul, Republic of Korea; ^2^ Graduate School of Biomedical Sciences, University of Texas Southwestern Medical Center, Dallas, TX, United States; ^3^ Department of Biomedical Engineering, Kyung Hee University, Yongin, Republic of Korea; ^4^ Department of Nuclear Medicine, Korea University College of Medicine, Seoul, Republic of Korea

**Keywords:** obesity, retinal vein occlusion, inflammation, visceral adipose tissue, positron emission tomography

## Abstract

**Background:**

Retinal vein occlusion (RVO) predominantly occurs in individuals over the age of 50, and obesity is a recognized risk factor for its development. The pro-inflammatory metabolic activity of visceral adipose tissue (VAT), which elevates systemic inflammation, is regarded as a key underlying mechanism contributing to the detrimental effects of obesity on atherosclerosis and hypercoagulability in RVO. In this study, we aimed to evaluate the inflammatory metabolic activity of VAT using ^18^F-fluorodeoxyglucose (FDG) positron emission tomography/computed tomography (PET/CT) and to examine its association with RVO.

**Material and methods:**

A total of 22 elderly patients with RVO (aged ≥ 50 years) who underwent ^18^F-FDG PET/CT for routine health screening, along with 15 age-matched control participants who also underwent ^18^F-FDG PET/CT for routine health screening, were enrolled. The metabolic activity of VAT was assessed using its maximum standardized uptake value (SUVmax), while systemic inflammation was evaluated based on the SUVmax of the spleen and bone marrow (BM), as well as C-reactive protein (CRP) levels.

**Results:**

The VAT SUVmax was higher in patients with RVO compared to the non-RVO group. Additionally, levels of systemic inflammation surrogate markers were elevated in patients with RVO relative to those without RVO. Furthermore, VAT SUVmax showed a positive correlation with systemic inflammation surrogate markers and was independently associated with the presence of RVO.

**Conclusions:**

The metabolic activity of VAT, as assessed by ^18^F-FDG PET/CT, was found to be associated with the presence of RVO and correlated with the degree of systemic inflammation. Therefore, VAT SUVmax may serve as a potential surrogate marker for obesity-related VAT inflammation linked to RVO.

## Introduction

Globally, retinal vein occlusion (RVO) is the second most common retinal vascular disease after diabetic retinopathy and is caused by partial or complete occlusion of the retinal vein (1). It predominantly occurs in individuals over the age of 50 (2) and is characterized by a disruption of normal blood flow due to venous blockage, which can lead to severe visual impairment and a detrimental impact on patients’ quality of life (3). Furthermore, it places a significant economic burden on the United States (2).

Extensive previous studies have suggested that obesity is a significant risk factor for RVO and with a consistent trend of increasing risk observed across all body mass index (BMI) quartile (4–6). Inflammation of visceral adipose tissue (VAT) is recognized as a key contributor to the adverse effects of obesity (7–9). Inflamed VAT, functioning as a metabolically active endocrine organ, secretes proinflammatory cytokines such as interleukin-6 (IL-6) and tumor necrosis factor-alpha (TNF-α) (7–9). These cytokines promote the infiltration of inflammatory cells, primarily macrophages, into the VAT, thereby exacerbating VAT inflammation (7–9).

Exacerbated inflammation of VAT promotes an increase in circulating systemic proinflammatory cytokines, thereby accelerating the progression of atherosclerosis (7–9). This process may lead to compression of adjacent veins, particularly at arteriovenous crossings, contributing to the development of RVO (10). Moreover, heightened VAT inflammation elevates circulating levels of clotting factors and suppresses the fibrinolytic pathway, collectively creating a hypercoagulable state in the retinal vein that may ultimately lead to the onset of RVO (11, 12).

Accumulating evidence indicates that ^18^F-fluorodeoxyglucose (FDG) positron emission tomography/computed tomography (PET/CT) serves as a reliable non-invasive imaging modality for evaluating the metabolic activity of VAT in humans (13–17). Moreover, a recent animal study demonstrated increased VAT metabolic activity, as assessed by ^18^F-FDG PET/CT, in an obesity mouse model, which correlated with elevated macrophage-driven inflammation within VAT (18). Clinical investigations have further revealed that heightened VAT metabolic activity is associated with greater tumor aggressiveness and the severity of coronary artery disease—both of which are conditions for which obesity, particularly inflamed VAT, is a recognized risk factor (13–15, 17). Based on these findings, we hypothesize that VAT metabolic activity may also be associated with RVO.

This study aimed to assess the inflammatory metabolic activity of VAT using ^18^F-FDG PET/CT and to investigate its association with RVO in elderly individuals undergoing routine health screening.

## Material and methods

### Study population

**Figure 1:**
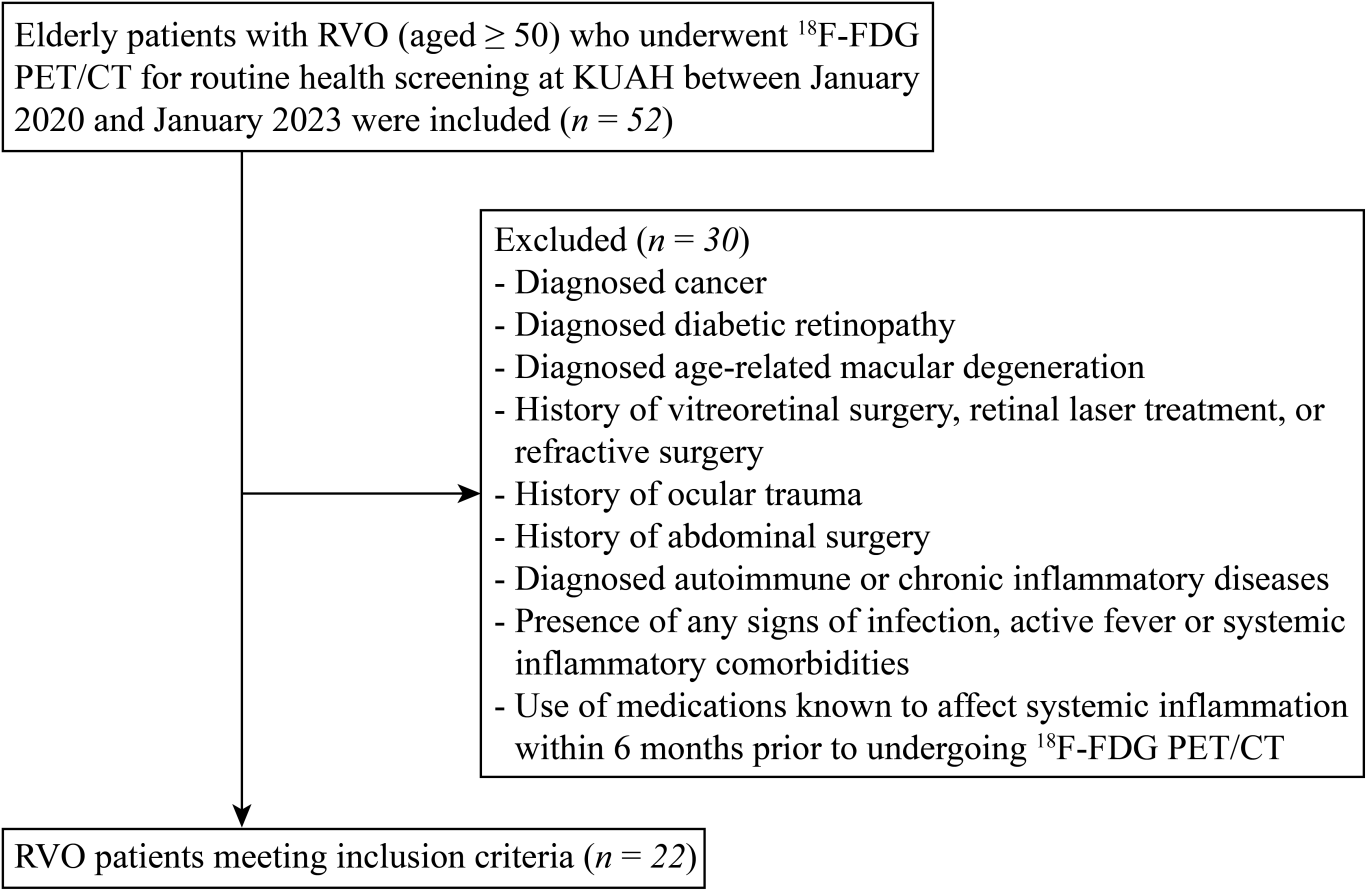
Flow diagram of the patient selection and enrollment process. ^18^F-FDG PET/CT, ^18^F-fluorodeoxyglucose (FDG) positron emission tomography/computed tomography (PET/CT); KUAH, Korea University Ansan Hospital.

Elderly patients with RVO (aged ≥ 50) who underwent ^18^F-FDG PET/CT for routine health screening at Korea University Ansan Hospital between January 2020 and January 2023 were included in this study (**Figure 1**). Patients were excluded if they had a diagnosis of cancer, diabetic retinopathy, or age-related macular degeneration. Exclusion criteria also included a history of vitreoretinal surgery, retinal laser treatment, refractive surgery, or ocular trauma. Patients with a history of abdominal surgery, diagnosed autoimmune or chronic inflammatory diseases, signs of infection, active fever, or systemic inflammatory comorbidities were also excluded. Additionally, the use of medications known to affect systemic inflammation within six months prior to undergoing ¹^8^F-FDG PET/CT resulted in exclusion. Finally, a total of 22 patients were included in this study. Additionally, 15 age-matched control participants who also underwent ^18^F-FDG PET/CT as part of routine health screening were enrolled.

This study was conducted in accordance with the principles of the Declaration of Helsinki and was approved by the Institutional Review Board of Korea University Ansan Hospital (Approval No. 2024AS0295). Owing to its retrospective design, the requirement for informed consent was waived by the Institutional Review Board.

### Anthropometric and laboratory measurements

Body mass index (BMI) was calculated as weight in kilograms divided by height in meters squared (kg/m²). All blood samples were collected following a 12-hour overnight fast. Serum levels of total cholesterol, triglycerides, and high-density lipoprotein (HDL) cholesterol were analyzed using an automated chemistry analyzer (Hitachi 747, Hitachi, Tokyo, Japan). Low-density lipoprotein (LDL) cholesterol concentrations were estimated using the Friedewald formula (19). High-sensitivity C-reactive protein (hsCRP) levels were determined using a chemiluminescence immunoassay (Beckman Coulter, Brea, CA, USA).

### ^18^F-FDG PET/CT protocol

All patients and participants underwent overnight fasting for more than 6 hours prior to the ^18^F-FDG PET/CT examination. Imaging commenced 60 minutes after intravenous administration of ^18^F-FDG at a dose of 5.29 MBq/kg, using a dedicated PET/CT scanner (Discovery 710, GE Healthcare, Milwaukee, WI, USA). The administered dose of ¹^8^F-FDG was in accordance with the guidelines of the Korean Society of Nuclear Medicine (20). Whole-body images were acquired from the skull vertex to the proximal thighs. A low-dose CT scan (120 kVp, 60 mA, 2.5 mm slice thickness) was first performed for attenuation correction, immediately followed by the PET acquisition. PET images were reconstructed using a three-dimensional ordered subset expectation maximization (3D-OSEM) algorithm with 2 iterations and 16 subsets.

### Image analysis

Image analysis was performed in a blinded manner with respect to clinical data, using a commercially available workstation (Advantage Workstation 4.6, GE Healthcare, Milwaukee, WI, USA). Initially, VAT and subcutaneous adipose tissue (SAT) were identified based on predefined Hounsfield unit thresholds (−70 to −110), as previously described (13–17). Regions of interest (ROIs) were then delineated, and the corresponding standardized uptake values (SUVs) were calculated using the following formula:


SUV = Tracer activity (ROI) (MBq/mL)/

Injected dose (MBq)/Total body weight (g)

To assess the metabolic activity of VAT, 10 ROIs were placed along the intra-abdominal fat boundaries across three consecutive axial slices at the L4–L5 vertebral levels. Care was taken to avoid inclusion of spillover uptake from adjacent structures such as the intestines, blood vessels, or muscles, in accordance with established protocols (13–17). VAT SUVmax was defined as the average of the maximum SUVs from these 10 ROIs.

Similarly, to evaluate the metabolic activity of SAT, 10 ROIs were positioned over the buttocks and the anterior abdominal wall across three consecutive axial slices at the same vertebral levels. SAT SUVmax was calculated as the average maximum SUV from these ROIs (13–17).

Increased uptake of ^18^F-FDG in the spleen and bone marrow (BM) is a well-established indicator of enhanced myelopoietic activity and is considered a surrogate marker of systemic inflammation (21, 22). For the evaluation of splenic and BM metabolic activity, ROIs were placed throughout the entire axial span of the spleen and across the L3–L5 vertebral bodies for the BM, respectively (21). The mean maximum SUV values from these ROIs were reported as spleen SUVmax and BM SUVmax.

### Statistical analysis

All data are expressed as mean ± standard deviation. Categorical variables were analyzed using the Chi-squared (χ²) test or Fisher’s exact test, as appropriate. The Shapiro–Wilk test was employed to assess the normality of continuous variables. For normally distributed data, comparisons were made using Student’s *t*-test, while the Mann–Whitney *U* test was applied to non-normally distributed data. Additional statistical analyses included Spearman’s rank correlation, receiver operating characteristic (ROC) curve analysis, and logistic regression. All statistical analyses were conducted using MedCalc software (version 18.5, MedCalc Software Ltd, Ostend, Belgium) and SPSS software (version 17.0, SPSS Inc, Chicago, IL, USA). A *p*-value < 0.05 was considered indicative of statistical significance.

## Results

Patients with RVO exhibited significantly higher rates of smoking and elevated systemic inflammatory markers compared to those without RVO. Clinical characteristics of all participants are summarized in **Table 1**.

**Table 1:** Clinical characteristics of all participants.

	Non-RVO	RVO	*p*
No. of patients	15	22	
Age (years)	63.8 ± 11.6	65.9 ± 8.9	0.66
Sex, n (%)			0.527
Male	7 (46.7)	14 (63.6)	
Female	8 (53.3)	8 (36.4)	
BMI (kg/m^2^)	23.4 ± 2.6	23.9 ± 3.6	0.667
HTN, n (%)			0.549
No	6 (40)	11 (50)	
Yes	9 (60)	11 (50)	
DM, n (%)			0.69
No	11 (73.3)	18 (81.8)	
Yes	4 (26.7)	4 (18.2)	
Cardiovascular disease, n (%)			1
No	13 (86.7)	19 (86.4)	
Yes	2 (13.3)	3 (13.6)	
Cerebral infarction, n (%)			0.368
No	14 (93.3)	17 (77.3)	
Yes	1 (6.7)	5 (22.7)	
Choronic kidney disease, n (%)			0.629
No	14 (93.3)	18 (81.8)	
Yes	1 (6.7)	4 (18.2)	
Glaucoma, n (%)			0.629
No	14 (93.3)	18 (81.8)	
Yes	1(6.7)	4 (18.2)	
Smoking, n (%)			0.037*
Never	10 (66.7)	7 (31.8)	
Ever	5 (33.3)	15 (68.2)	
Alcohol, n (%)			0.481
Never	12 (80)	15 (68.2)	
Ever	3 (20)	7 (31.8)	
Cataract surgery, n (%)			0.609
No	9 (60)	15 (68.2)	
Yes	6 (40)	7 (31.8)	
Intraocular pressure, mmHg	14.9 ± 2.9	15 ± 2	0.824
Ocular perfusion pressure, mmHg	47.5 ± 6.7	48.1 ± 10.8	0.8346
Total cholesterol, mg/dL	180.2 ± 52.9	177.9 ± 53.4	1
Triglycerides, mg/dL	134.3 ± 99.4	138.4 ± 75.7	0.568
HDL cholesterol, mg/dL	53.8 ± 19.6	48.3 ± 13.2	0.363
LDL cholesterol, mg/dL	92 ± 37.4	95.7 ± 43.4	0.865
hsCRP, mg/dL	0.4 ± 0.6	2.4 ± 3.3	0.013*
Spleen SUVmax	1.3 ± 0.1	1.8 ± 0.4	<0.001*
BM SUVmax	1.26 ± 0.1	1.7 ± 0.3	<0.001*

*RVO*, retinal vein occlusion; *BMI*, body mass index; *HTN*, hypertension; *DM*, diabetes mellitus; *HDL*, high-density lipoprotein; *LDL*, low-density lipoprotein; *hsCRP*, high-sensitivity C-reactive protein; *SUVmax*, maximum standardized uptake value; *BM*, bone marrow.

* Statistically significant difference.

### Increased VAT metabolic activity in RVO

**Figure 2:**
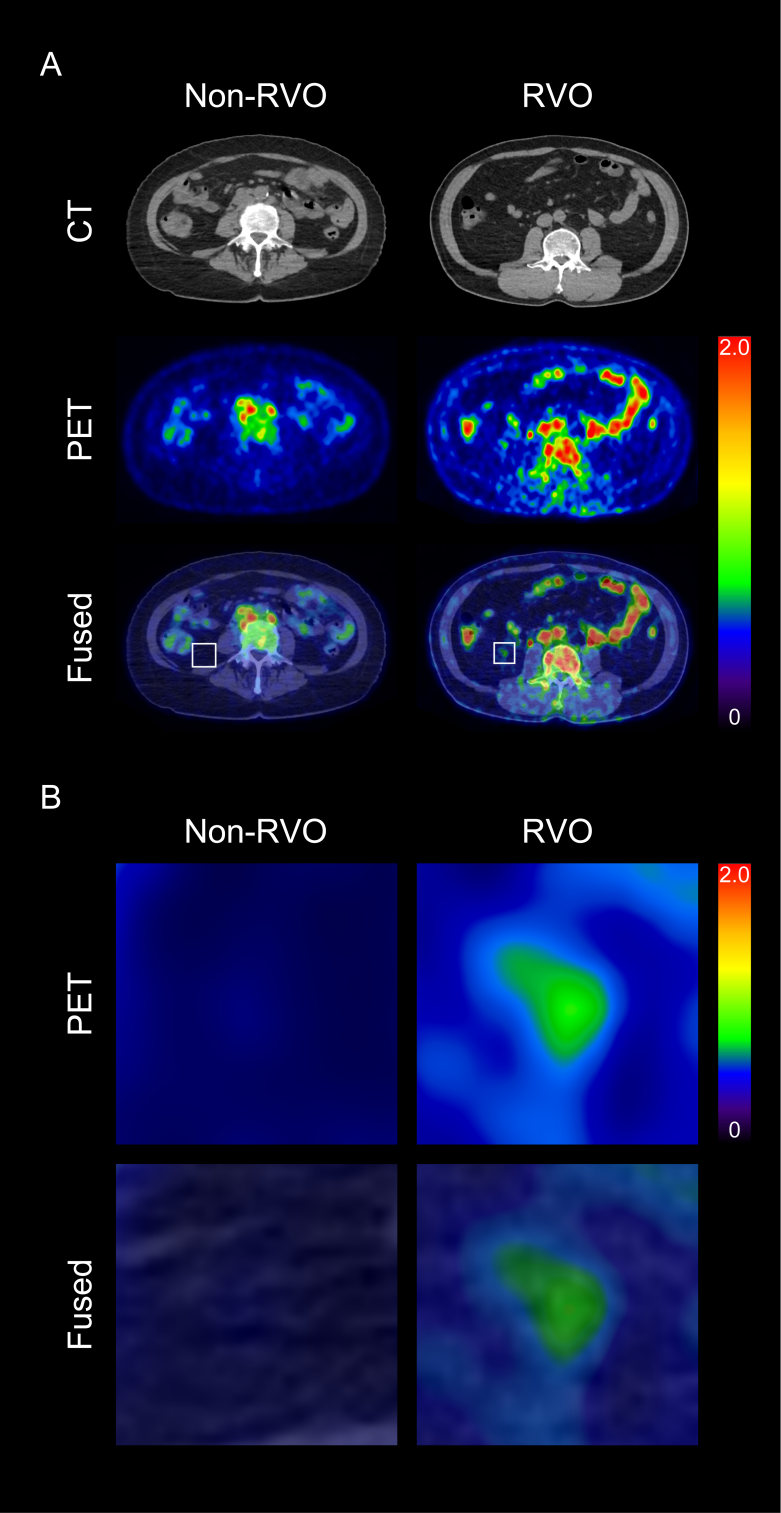
Representative images of visceral adipose tissue (VAT) metabolic activity based on the presence of retinal vein occlusion (RVO) **(A)**, and corresponding magnified views **(B)** CT, computed tomography; PET, positron emission tomography.

**Figure 3:**
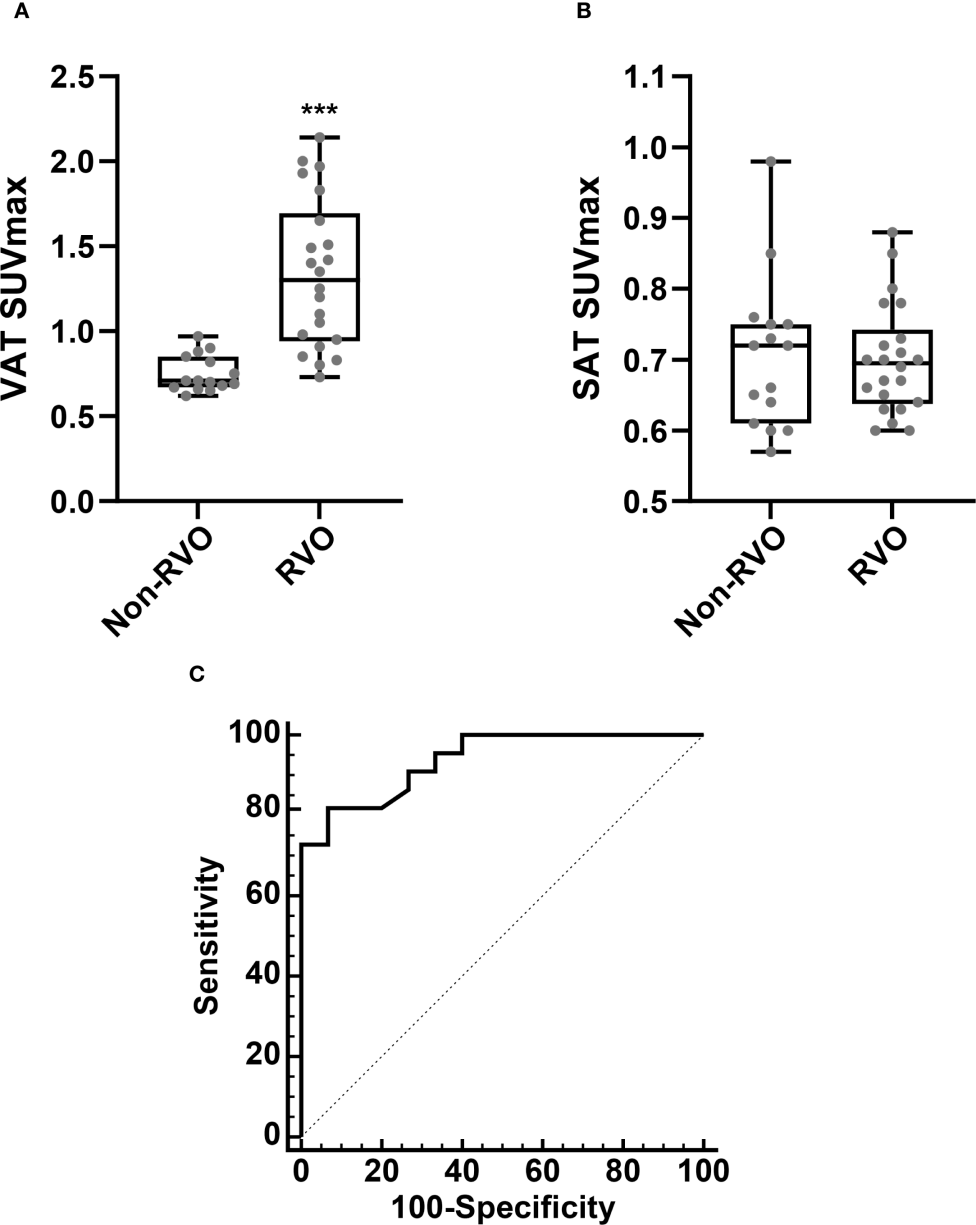
Comparison of VAT SUVmax **(A)** and SAT SUVmax **(B)**, based on the presence of RVO. Receiver operating characteristic (ROC) curve analysis for the identification of RVO **(C)**. Non-RVO, *n* = 15; RVO *n* = 22. SUVmax, standardized uptake value; SAT, subcutaneous adipose tissue. ***p < 0.001.

We first assessed whether VAT metabolic activity differed between groups. As shown in **Figures 2** and **3A**, VAT SUVmax was significantly higher in the RVO group than in the non-RVO group (1.33 ± 0.44 vs. 0.75 ± 0.10, *p* < 0.001). In contrast, SAT SUVmax did not differ significantly between groups (0.70 ± 0.08 vs. 0.70 ± 0.10, *p* = 1; **Figure 3B**).

### Association between VAT metabolic activity and systemic inflammation

Next, we investigated the relationship between VAT metabolic activity and systemic inflammation in RVO patients. As presented in **Table 1**, markers of systemic inflammation—including hsCRP, spleen SUVmax, and BM SUVmax—were significantly elevated in the RVO group. Correlation analysis revealed significant positive associations between VAT SUVmax and each inflammatory marker. In contrast, SAT SUVmax showed no significant correlation with systemic inflammation (**Table 2**).

**Table 2:** Spearman’s rank correlation analysis.

	VAT SUVmax		SAT SUVmax	
	*r*	*p*	*r*	*p*
Spleen SUVmax	0.785	<0.001*	0.217	0.196
BM SUVmax	0.823	<0.001*	0.161	0.342
hsCRP	0.824	<0.001*	0.097	0.592

*VAT*, visceral adipose tissue; *SAT*, subcutaneous adipose tissue; *SUVmax*, maximum standardized uptake value; *BM*, bone marrow; *hsCRP*, high-sensitivity C-reactive protein.

* Statistically significant difference.

### VAT metabolic activity is independently associated with RVO

To evaluate the association between VAT metabolic activity and RVO, we conducted a ROC curve analysis to determine the optimal cut-off value of VAT SUVmax for identifying RVO. As illustrated in **Figure 3C**, the optimal VAT SUVmax threshold was 0.9, yielding a sensitivity of 81.8% and a specificity of 93.3%. The area under the curve (AUC) was 0.938 (95% confidence interval: 0.807–0.991; standard error: 0.04; *p* < 0.001).

Subsequently, univariate and multivariate logistic regression analyses were conducted to evaluate the association between VAT SUVmax and RVO, using the optimal cut-off value of VAT SUVmax. In the univariate analysis, a history of smoking and elevated VAT SUVmax were significantly associated with RVO (**Table 3**). In the multivariate analysis, increased VAT SUVmax remained independently associated with RVO and demonstrated the highest odds ratio among all variables analyzed (**Table 3**).

**Table 3:** Uni- and multivariate analyses for the prediction of RVO.

Variable	Univariate		Multivariate	
	OR (95% CI)	*p*	OR (95% CI)	*p*
Age (Continuous)	1.015 (0.952 – 1.082)	0.651		
Sex (Female vs Male)	2 (0.526 – 7.604)	0.309		
BMI (Continuous)	1.049 (0.849 – 1.296)	0.657		
HTN (No vs Yes)	0.667 (0.177 – 2.517)	0.55		
DM (No vs Yes)	0.611 (0.126 – 2.955)	0.54		
Cardiovascular disease (No vs Yes)	1.026 (0.15 – 7.023)	0.979		
Cerebral infarction (No vs Yes)	4.118 (0.429 – 39.482)	0.22		
Chronic kidney disease (No vs Yes)	3.111 (0.312 – 31.028)	0.333		
Glaucoma (No vs Yes)	3.111 (0.312 – 31.028)	0.333		
Smoking (Never vs Ever)	4.286 (1.058 – 17.363)	0.041*	3.163 (0.427 – 23.42)	0.26
Alcohol (Never vs Ever)	1.876 (0.396 – 8.803)	0.43		
Cataract surgery (No vs Yes)	0.7 (0.178 – 2.75)	0.609		
Intraocular pressure (Continuous)	1.034 (0.778 – 1.373)	0.818		
Ocular perfusion pressure (Continuous)	1.008 (0.938 – 1.084)	0.829		
Total cholesterol (Continuous)	0.999 (0.985 – 1.013)	0.905		
Triglycerides (Continuous)	1.001 (0.992 – 1.009)	0.89		
HDL cholesterol (Continuous)	0.978 (0.934 – 1.025)	0.355		
LDL cholesterol (Continuous)	1.002 (0.984 – 1.021)	0.805		
VAT SUVmax (≤0.9 vs >0.9)	63 (6.317 – 628.323)	<0.001*	56.162 (5.409 – 583.098)	0.001*
SAT SUVmax (Continuous)	0.467 (0 – 748.049)	0.84		

*OR*, odds ratio; *CI*, confidence interval; *BMI*, body mass index; *HTN*, hypertension; *DM*, diabetes mellitus; *HDL*, high-density lipoprotein; *LDL*, low-density lipoprotein; *VAT*, visceral adipose tissue; *SAT*, subcutaneous adipose tissue; *SUVmax*, maximum standardized uptake value.

* Statistically significant difference.

## Discussion

To the best of our knowledge, this is the first human study to examine the association between VAT metabolic activity and RVO in an elderly population using ¹^8^F-FDG PET/CT imaging. Our findings demonstrate that increased VAT metabolic activity, as measured by ¹^8^F-FDG PET/CT, is significantly associated with RVO. Patients with RVO exhibited elevated VAT SUVmax, higher systemic inflammatory markers, and a strong correlation between VAT metabolic activity and systemic inflammation. Notably, VAT SUVmax remained independently associated with RVO after adjusting for other known risk factors, including smoking.

These results provide novel mechanistic insight into the role of VAT inflammation in the pathogenesis of RVO. While previous studies have shown that VAT-derived proinflammatory cytokines contribute to systemic inflammation and atherosclerosis (7–9), our study offers direct *in vivo* evidence linking increased VAT metabolic activity to RVO. Inflammation within VAT likely promotes endothelial dysfunction and vascular remodeling through the release of circulating cytokines, particularly at arteriovenous crossings in the retina—an anatomical site susceptible to venous compression and occlusion (7–10). This localized mechanical stress, when compounded by systemic inflammation, may impair venous outflow and contribute to RVO development. Furthermore, the strong correlation between VAT SUVmax and systemic inflammatory markers suggests that VAT inflammation fosters a prothrombotic state. Increased clotting factors and reduced fibrinolytic activity, both associated with VAT inflammation, may further elevate the risk of retinal vein thrombosis (11, 12). The absence of a similar association with subcutaneous adipose tissue metabolic activity underscores the unique pathogenic role of visceral fat in systemic inflammation and vascular complications.

In addition to VAT inflammation, we also examined traditional risk factors. Smoking was significantly associated with RVO, consistent with previous meta-analyses (23). However, no significant association was observed between BMI and RVO. Although BMI is a widely used and easily accessible measure of obesity, it is a crude anthropometric index that does not reflect underlying metabolic dysfunction (15, 24). In particular, BMI fails to capture the inflammatory activity of VAT, which is increasingly recognized as a key contributor to obesity-related complications (25, 26). In contrast, ¹^8^F-FDG PET/CT imaging allows for the noninvasive assessment of VAT metabolic activity by detecting increased glucose uptake in activated macrophages, the predominant inflammatory cell type within inflamed VAT (18). Taken together, these findings suggest that VAT SUVmax may serve as a more specific surrogate marker of VAT-associated inflammation relevant to RVO pathogenesis, offering greater predictive value than BMI alone.

These findings have important clinical implications. The identification of VAT metabolic activity as an independent factor associated with RVO highlights the potential role of VAT metabolic inflammation in retinal vascular disease. As PET/CT imaging becomes increasingly integrated into routine health evaluations—particularly in elderly populations—VAT SUVmax could provide an opportunity for early identification of individuals at elevated risk for retinal vascular events. Incorporating VAT metabolic profiling into cardiovascular and ophthalmologic risk assessments may enable earlier, targeted interventions. Moreover, this study supports the broader notion that therapeutic strategies aimed at reducing VAT inflammation—through lifestyle modifications, pharmacologic treatments, or metabolic monitoring—could be beneficial in preventing both systemic and retinal vascular diseases. Future research should investigate whether reducing VAT inflammation lowers the incidence or recurrence of RVO in high-risk populations.

This study has several limitations. First, it was a retrospective, single-center investigation, which may introduce selection bias. A larger, prospective, multicenter study is warranted to validate and expand upon these findings. Second, we were unable to control all lifestyle factors—such as physical activity and dietary patterns, including the intake of high-glycemic, refined, and processed foods—which may influence RVO development. Third, although ¹^8^F-FDG PET/CT is a validated modality for assessing VAT metabolic activity, we did not perform histological validation using VAT tissue samples, which could have confirmed the presence of inflammatory cell infiltration. Finally, we could not fully account for all variables affecting FDG uptake, including plasma glucose and insulin levels, or the interval between tracer injection and image acquisition. Nevertheless, the use of robust, noninvasive imaging technique enabled a novel assessment of VAT inflammation in relation to RVO, mitigating some of these limitations and offering important new insights.

In conclusion, this study demonstrated that increased metabolic activity of VAT, as measured by ¹^8^F-FDG PET/CT, is independently associated with RVO in an elderly population. These findings provide *in vivo* evidence linking VAT inflammation to retinal vascular pathology and underscore its potential role as a mechanistic contributor beyond traditional risk factors such as BMI. The observed association between VAT SUVmax and systemic inflammatory markers further supports the contribution of VAT-driven inflammation to a prothrombotic state conducive to RVO. Collectively, these results suggest that metabolic profiling of VAT may serve as a valuable biomarker for risk stratification and early detection of RVO. Future prospective studies are warranted to validate these findings and to investigate whether therapeutic modulation of VAT inflammation can reduce the incidence or recurrence of retinal vascular disease.

## Data Availability

The original contributions presented in the study are included in the article/supplementary material. Further inquiries can be directed to the corresponding authors.
